# Phylogenomic Analysis of *Campylobacter fetus* Reveals a Clonal Structure of Insertion Element IS*Cfe1* Positive Genomes

**DOI:** 10.3389/fmicb.2020.585374

**Published:** 2020-11-12

**Authors:** Mostafa Y. Abdel-Glil, Helmut Hotzel, Herbert Tomaso, Jörg Linde

**Affiliations:** ^1^Institute of Bacterial Infections and Zoonoses, Friedrich-Loeffler-Institut, Jena, Germany; ^2^Department of Pathology, Faculty of Veterinary Medicine, Zagazig University, Ash Sharqiyah, Egypt

**Keywords:** *Campylobacter fetus*, *ISCfe1*, bovine genital campylobacteriosis, WGS, MLST

## Abstract

Subspecies of the species *Campylobacter fetus* are associated with specific host niches including mammals and reptiles. *Campylobacter fetus* subsp. *fetus* is a zoonotic pathogen infecting humans. Infections can vary from an acute intestinal illness to severe systemic infections, with sheep and cattle as major reservoirs. In contrast, *Campylobacter fetus* subsp. *venerealis* causes bovine genital campylobacteriosis, which leads to abortion in cattle and a high economic burden for the farmers. Therefore, high-quality molecular subtyping is indispensable for interventional epidemiology. We used whole-genome sequencing (WGS) data of 283 *Campylobacter fetus* strains from 18 countries and compared several methods for *Campylobacter fetus* subtyping, including WGS, multilocus sequence typing, PCR assays, and the presence of the insertion element IS*Cfe1*. We identified a highly clonal clade (designated as clade 1) that harbors the insertion sequence IS*Cfe1*. The presence of this insertion sequence is an essential diagnostic tool for the identification of the subspecies *Campylobacter fetus* subsp. *venerealis*, serving as a target for several PCR assays. However, we have found a high sequence variability for the IS*Cfe1* besides the presence of IS*Cfe1*-paralogues in certain other genomes (*n* = 7) which may cause incorrect diagnostic results. Clade 1 seems to be the cattle-specific clade of this species. We propose that only this clade might be designated as *Campylobacter fetus* subsp. *venerealis* as it harbors the IS*Cfe1* marker sequence, which is a major target for molecular methods currently used for *Campylobacter fetus* subspecies identification. Fostering this proposal, we defined eleven stable nucleotide markers specific for this clade. Additionally, we developed a bioinformatics toolbox for the fast identification of this clade based on WGS data. In conclusion, our results demonstrate that WGS can be used for *Campylobacter fetus* subtyping overcoming limitations of current PCR and MLST protocols.

## Introduction

The species *Campylobacter* (*C.*) *fetus* comprises three subspecies with distinct clinical significance: *C. fetus* subsp. *fetus* (*Cff*) and *C. fetus* subsp. *venerealis* (*Cfv*) are both mammal-associated, and *C. fetus* subsp. *testudinum* (*Cft*) occurs in reptiles (Gilbert et al., 2017, 2018). Strains of *Cfv* are highly niche-specific and restricted to the genital tract of cattle (Silveira et al., 2018). *Cfv* causes bovine genital campylobacteriosis (BGC), a disease associated with abortion and infertility in cattle with significant economic losses (Silveira et al., 2018). In contrast, *Cff* was found in humans, cattle, sheep, and other animal species. *Cff* resides as normal flora in the human’s intestine (Sprenger et al., 2012; Iraola et al., 2017) but can also cause diarrhea or systemic disease in humans and sporadic abortion in sheep and cattle (Guillermo et al., 2001; Chaillon et al., 2010; Sprenger et al., 2012; Iraola et al., 2017).

Given the distinct host-niche preference and clinical relevance, subspecies differentiation is crucial for disease control and veterinary public health (Schulze et al., 2006). The reptile-associated *Cft* is divergent from *Cfv* and *Cff* (Fitzgerald et al., 2014) with pairwise average nucleotide identity (ANI) of ∼92% (Iraola et al., 2017), which is below the commonly accepted ANI for species definition (95%) (Richter and Rossello-Mora, 2009). Therefore, *Cft* was proposed as an independent species (Iraola et al., 2017). *Cfv* and *Cff*, on the other hand, are very similar at the DNA level (Iraola et al., 2017). These subspecies can be distinguished using biochemical testing based on 1% glycine tolerance and H_2_S production in cysteine media (Schulze et al., 2006; Silveira et al., 2018). However, these methods have poor reproducibility and are usually not concordant with molecular methods (Van Der Graaf-Van Bloois et al., 2014). Molecular methods for *C. fetus* subtyping include multilocus sequence typing (MLST) (Van Bergen et al., 2005a), amplified fragment length polymorphisms (AFLP) (Van Bergen et al., 2005b), pulsed-field gel electrophoresis (PFGE) (On and Harrington, 2001), and polymerase chain reaction (PCR) (Wang et al., 2002; Abril et al., 2007; Mcgoldrick et al., 2013; Van Der Graaf-Van Bloois et al., 2013). The latter is valuable for detecting *Cfv* without culturing and includes *Cfv*-specific PCRs for detecting IS*Cfe1* (Abril et al., 2007; Mcgoldrick et al., 2013; Van Der Graaf-Van Bloois et al., 2013), a *parA* gene (Hum et al., 1997; Mcmillen et al., 2006) or others targets (Moolhuijzen et al., 2009; Iraola et al., 2012), but also a *Cff*-specific PCR (Wang et al., 2002).

Germany is currently declared free of bovine genital campylobacteriosis. Nevertheless, each bull used for semen production has to be tested once a year for the presence of *C. fetus* subsp. *venerealis* and *Tritrichomonas foetus*. Only if both tests are negative, the semen may be sold. The present diagnostic methods used for subspecies detection of *C. fetus* lack reproducibility or are sometimes contradictory. Therefore, this study aimed at using public genomic data of 283 *C. fetus* strains and *in silico* comparing molecular methods used to assign the *C. fetus* subspecies. We further identified new nucleotide markers and developed a bioinformatics tool for *C. fetus* subtyping using WGS for outbreak investigations.

## Materials and Methods

### Retrieval and Curation of Public Genomic Data

**FIGURE 1:**
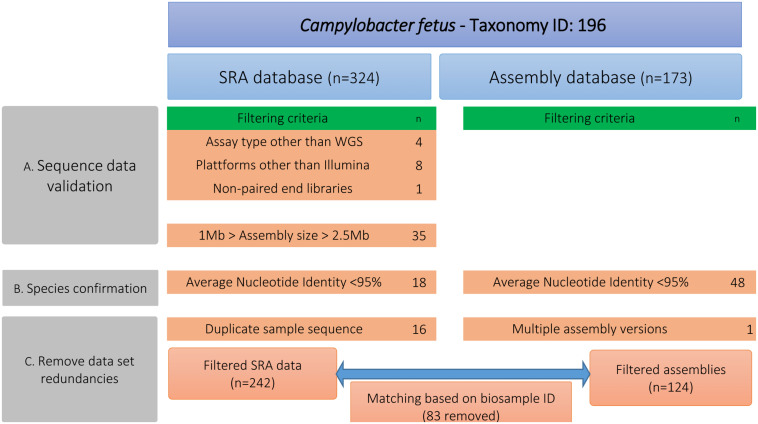
Different steps for filtering the publicly available genomic data of *Campylobacter fetus* including number of filtered data sets. Step A performed quality assurance via removing genomes that deviated from the average *C. fetus* genome size (∼1.8 Mbp ± 25%). Step B measured pairwise ANI and excluded genomes with <95% accordance. Step C removed data redundancies by matching raw sequence data and genome assemblies based on the BioSample-ID.

We downloaded publicly available genomes of *C. fetus* from NCBI, including assemblies (*n* = 173) and raw reads (*n* = 327) (Supplementary Table S1). In a first step (A), these public data were curated for quality assurance via removing outlier genomes (Figure 1). We excluded genomes that deviated from the average *C. fetus* genome size (∼1.8 Mbp ± 25%), possibly indicating contamination or insufficient DNA sequencing quality. For that, raw reads were assembled using shovill v1.0.4^1^ (options –minlen 500 –mincov 3). Step B performed species confirmation via measuring pairwise ANI using pyani v0.2.3 (Pritchard et al., 2016) and excluded genomes with < 95% accordance (Supplementary Table S2). This procedure removed all *Cft* genomes. Step C removed data redundancies via matching sequences from SRA and assembly databases based on BioSample-ID. In the case that samples were available in both databases, we kept only raw reads. The final data set comprised 283 genomes (Table 1, details Supplementary Table S1). These strains are geographically diverse as they were collected from 18 countries of six continents. Isolates from Europe were the most frequent (41%) followed by those from North America (32%) and South America (14%). The isolates were collected between 1952 and 2018. Many isolates were of bovine origin (37%) reflecting the preferred ecological niche of this species, followed by human (26%) and ovine isolates (6%) as well as one isolate from a monkey and two from red-footed tortoises (*Chelonoidis carbonarius*) (Gilbert et al., 2017, 2018; Iraola et al., 2017).

**TABLE 1:** Summary of metadata of 283 *Campylobacter fetus* strains downloaded from public repositories and analyzed in the study.

Isolation geographic source			Isolation time range		Origin of isolation (No.)				
Continent	Country of isolation	n. (%)	From – To	Missing	Bovine	Human	Ovine	Others	Missing
South America		40 (14.1)							
	Argentina	35 (12.4)	1989 – 2015	4	35				
	Uruguay	5 (1.8)	2013 – 2017		1	3	1		
North America		92 (32.5)							
	Canada	16 (5.7)	2005 – 2014	–	4	12			
	United States	76 (26.9)	2009 – 2018	65	2	1			73
Europe		117 (41.3)							
	Belgium	1 (0.4)		1	1				
	Czech Republic	1 (0.4)		-					1
	France	39 (14.8)	2004 – 2014	1	1	38			
	Germany	19 (6.7)	1999 –2014	-	16		2	Monkey (1)	
	Ireland	1 (0.4)	2017			1			
	Netherlands	3 (1.1)	2000 – 2012		1			*Chelonoidis carbonarius* (2)	
	United Kingdom	22 (7.8)	1952 – 2015	3	9		12		1
	Spain	31 (10.9)	2002 – 2014		31				
Asia		19 (6.7)							
	India	1 (0.4)	2013			1			
	Taiwan	17 (6)	2002 – 2014			17			
	Turkey	1 (0.4)	2013			1			
Oceania		4 (1.4)							
	New Zealand	1 (0.4)	2014				1		
	Australia	3 (1.1)	1964 – 1967	1	2				1
Africa		2 (0.7)							
	South Africa	2 (0.7)	2006 – 2007		2				
Missing		9 (3.2)				1	2		6
Total		283			105	75	18	3	82

### Whole Genome-Based Bioinformatics Analysis

For whole-genome comparison, we used snippy v4.6^2^ to identify high-quality variants with Illumina reads as input and the genome of strain 82–40 (accession CP000487) as a reference. The default parameters of snippy v4.6 were applied. Assembled genomes were included using the option (–ctgs) in snippy v4.6. For genome clustering, we used hierBAPS (Cheng et al., 2013), which defines clades hierarchically independent from a phylogenetic analysis. As input for hierBAPS, we used recombination filtered and unfiltered core genome alignment. The MATLAB version of hierBAPS was used with two nested clustering levels, and *a priori* upper bound for clusters number set over the interval between 10 and 100 for 10 independent runs. Gubbins v2.2.1 was used with default settings (Croucher et al., 2015) to remove recombination sites. As input for Gubbins, we constructed a pseudo-whole-genome alignment using snippy-clean_full_aln^2^ in which the strains’ genome sequences were replaced by the reference genome updated with specific SNPs for each strain (Croucher et al., 2015). The number of SNP variations between genome pairs were calculated using snp-dists v0.6.2^3^. Consensus SNP sites were extracted using snp-sites v2.4.1^4^. To root the phylogenetic tree, we initially included a *Cft* genome (accession CP027287) and identified two *Cff* genomes from reptiles that were divergent to the data set. RAxML v8.2.10 (Stamatakis, 2014) was used to construct a maximum likelihood (ML) phylogeny using the GTR + Γ nucleotide substitution model, and 100 bootstrap support for bipartitions (options -m ASC_GTRGAMMA –asc-corr = lewis). The ML tree was visualized using iTOL (Letunic and Bork, 2019) and FigTree v1.4.3^5^.

We used whole-genome data to predict MLST types, PCR amplicons, and insertion elements. MLST profiles were determined based on PubMLST database (Jolley and Maiden, 2010) using mlst v2.15.2^6^ and ARIBA v2.14.4 (Hunt et al., 2017) with default settings. For *in silico* PCR, we used a PERL script^7^ (accessed 11.2019, default settings) and Geneious prime 2019.2.3^8^ with primer sets from previous studies (Hum et al., 1997; Mcmillen et al., 2006; Abril et al., 2007; Mcgoldrick et al., 2013; Van Der Graaf-Van Bloois et al., 2013; Iraola et al., 2016; Supplementary Table S3). Looking for IS elements, we used ISEscan v1.5.4 (Xie and Tang, 2017) to detect IS families. We additionally used the software abricate^9^ to search the genomes through BLAST (options 90% identity and 30% overlap) for the presence of IS element “IS*Cfe1*” from the IS finder database. The IS*Cfe1* paralogues were identified based on the detection of multiple BLAST hits with full coverage in the genomes. To show the differences in the location of IS*Cfe1* paralogues across the chromosomes of the strains, we used progressiveMauve (Darling et al., 2010) for multiple genome alignment with annotated FASTA files as input. This analysis was restricted to the complete circularized genomes (*n* = 6 strains) of clade 1 in which multiple copies of IS*Cfe1* were detected. For sequence analysis of IS*Cfe1*, we firstly parsed the output of abricate^9^ to extract IS*Cfe1* sequences longer than 1,500 bp. The software MAFFT v7.307 (Katoh and Standley, 2013) aligned the extracted sequences with options (–auto –adjustdirection). We then used MEGA X (Kumar et al., 2018) to report IS*Cfe1* sequence divergence with all ambiguous positions being removed for each sequence pair (pairwise deletion option). The average nucleotide differences over sequence pairs within and between the IS*Cfe1* sequence groups were determined using MEGA X (Kumar et al., 2018) with options “compute within/between group mean distance.” To identify strict core genes and gene variants in different clades, we used the software Ridom Seqsphere+ v5.1.0 (Junemann et al., 2013) with default settings. The identified genes were tested for the presence of recombination using the Pairwise Homoplasy Index (PHI) statistic (Bruen et al., 2006), as implemented in the PHIPack software (Bruen, 2005).

For the evaluation of the bioinformatics tool “*cfvCatch*,” we assessed its reproducibility using 20 randomly selected samples at different sequencing depths. For that, we used seqtk^10^ to *in silico* subsample Illumina read sets of the 20 randomly selected *C. fetus* strains at sequencing depths ranging between 10× and 100×. The resulting read sets were used as input for the *cfvCatch*. Statistical values of the assembled genomes were reported using SeqKit (Shen et al., 2016).

## Results and Discussion

**FIGURE 2:**
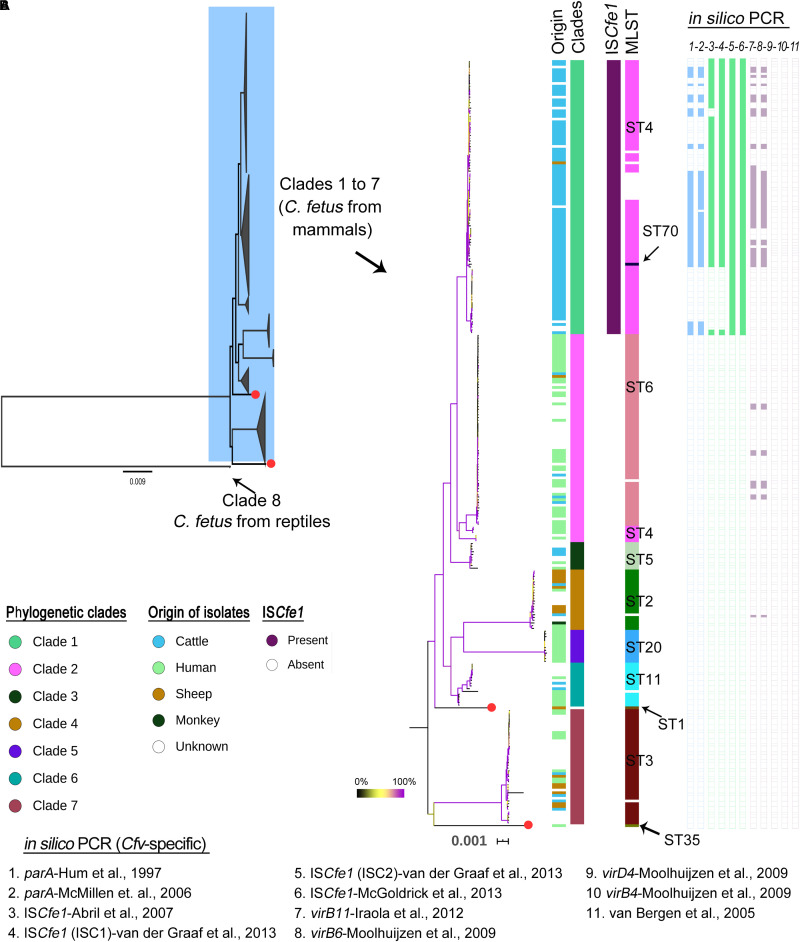
Phylogenetic structure of *Campylobacter fetus* genomes based on 18,793 high-quality SNPs that were present in a clonal frame in the 281 *C. fetus* genomes. **(A)** A maximum likelihood phylogenetic tree with the eight major clades of the species being collapsed. Red colored tips indicate two strains regarded as singletons. Note that clade 8 includes two *Campylobacter fetus* genomes from reptiles which were divergent to mammalian *C. fetus* genomes (ANI ∼97.8%). **(B)** A maximum likelihood phylogenetic tree showing clades 1 to 7. The bootstrap values are indicated with branch coloring according to the legend provided in the figure, 0 to 100% bootstrap support corresponds to the branch colors from black to violet, using yellow as the midpoint. Columns beside the phylogenetic tree correspond, respectively, to host of isolation, phylogenetic clades, the presence of IS*Cfe1* element, the MLST types, and the results of *in silico* PCR (numbered one to eleven according to the primer used as in legends). White spaces in the PCR indicate negative results while in MLST, they indicate no assignment to known MLST type.

A recent study by Iraola et al. (2017) classified a global collection of 182 *C. fetus* strains into eight phylogenetic lineages. The data of Iraola et al., (*n* = 169 out of 182 genomes) were combined with 114 additional genomes from independent studies as well as sequence data from the NCBI (Table 1, details Supplementary Table S1). Based on this extended data set (*n* = 283 strains), we identified 18,793 high-quality SNPs that were present in a clonal frame (non-recombinant SNPs) in 281 genomes, while two genomes (GCF_000174675 [Azul-94] and SRR6377517 [PNUSAC001504]) were unsuitable for recombination and phylogenetic analysis because of the high percentage of missing/ambiguous nucleotides (>25%). Two reptilian *C. fetus* were very distant to other *C. fetus* from mammals with average ANI ∼97.8%. In contrast, mammalian *C. fetus* harbored highly homogenous genomes with >99.2% nucleotide similarity (Supplementary Table S2 and Figure 2). Based on SNP architecture in the core genome of the strains, we concluded the clade structure in a phylogeny-dependent (using a maximum likelihood tree) and independent manner (with hierBAPS). The 281 genomes were classified into eight clades including 279 genomes. In addition, two genomes were assigned as singletons (ERR976359 [2006 479 h] and GCF_007723545 [17144]) (Figure 2 and Supplementary Table S3). The size of the eight clades varied between two and 100 genomes and the number of SNP sites detected for the clades were as follow: 687 SNP sites in clade 1 [*n* = 100 genomes], 865 SNP sites in clade 2 [*n* = 76 genomes], 226 SNP sites in clade 3 [*n* = 10 genomes], 352 SNP sites in clade 4 [*n* = 22 genomes], 112 SNP sites in clade 5 [*n* = 12 genomes], 444 SNP sites in clade 6 [*n* = 16 genomes], 548 SNP sites in clade 7 [*n* = 42 genomes] and 3 SNP sites in clade 8 [*n* = 2 genomes]. These results were in good congruence with previous studies. However, we used a larger data set and a different methodology to report core genome SNPs, confirming the high clonality of *C. fetus* strains. In addition, clade 1 strains (as found in this and previous studies) were restricted to cattle except one strain from a sheep and ten strains of unknown origin (Figure 2). The other clades (2–8) comprised strains isolated from different hosts, including humans, sheep, cattle, and others (Figure 2). Using this global reference phylogeny, we *in silico* compared molecular methods used for the subtyping of *C. fetus.*

### The Presence of the IS*Cfe1* Insertion Sequence and Its Sequence Variability

**FIGURE 3:**
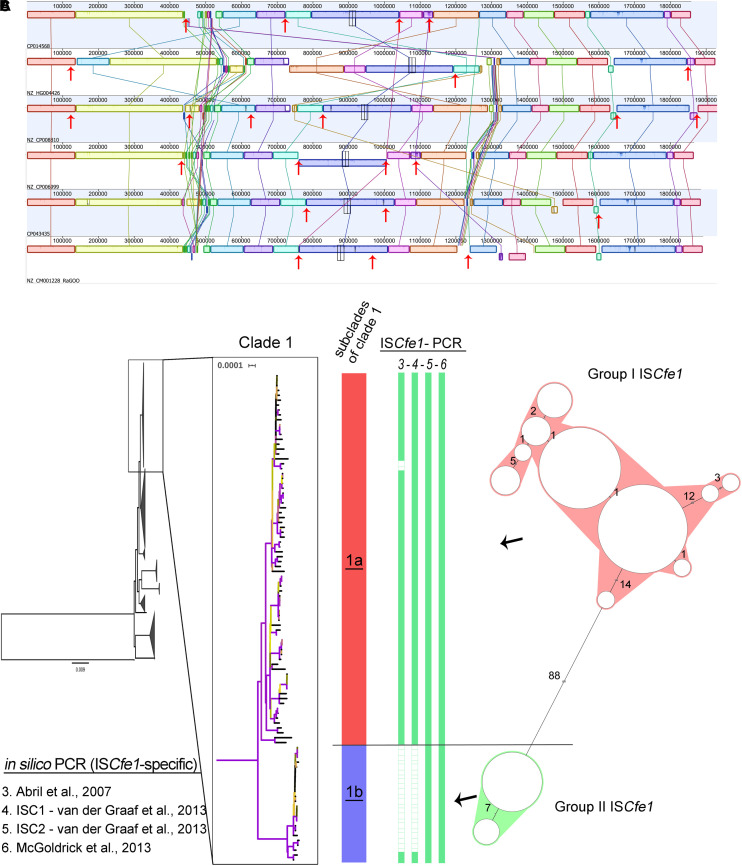
The presence of the IS*Cfe1* insertion sequence and its sequence variability. **(A)** Mauve alignment of the chromosomes from the six complete *Campylobacter fetus* genomes in clade 1 showing the location of the IS*Cfe1* paralogues across the chromosomes (red arrows) and their frequent association with inversions and shifts of the chromosomal locally collinear blocks. **(B)** A close-up view of the phylogenetic clade 1 shows two distinct subclades (1a and 1b). Two IS*Cfe1* groups based on the cluster analysis of the IS*Cfe1* sequences **(C)** were reflected by the phylogenetic structure of clade 1. Group I IS*Cfe1* is present in the subclade “1a” while group II of IS*Cfe1* is present in subclade “1b”.

IS*Cfe1* has been reported as a specific marker for *Cfv* (Abril et al., 2007; Mcgoldrick et al., 2013). We searched the genomes through BLAST for the IS*Cfe1* with thresholds: 90% identity and 30% overlap. The results showed that this marker element exists exclusively in all clade 1 genomes (Figures 2, 3).

Further analysis of the IS*Cfe1* identified the presence of multiple copies (IS*Cfe1* paralogues) in certain genomes comprising the six complete genomes in clade 1 (Figure 3A) as well as one draft genome. Three to eight copies per genome were present (Supplementary Tables S3, S4). In the complete genomes, the IS*Cfe1* paralogues were distributed on the strains’ chromosome (3 – 6 copies per chromosome) and plasmids (1 – 3 copies per plasmid; Supplementary Table S4). The average identity of the IS*Cfe1* paralogues within the same genome varied between 98 and 100% (Supplementary Table S4). The positions of the IS*Cfe1* across the chromosomes are shown in Figure 3A. Interestingly, IS*Cfe1* was located at the boundaries of several locally collinear blocks in the chromosome. Some inversions of the chromosomal blocks were also bordered by this insertion sequence.

It is noteworthy to mention that the failed detection of the ISCfe1 paralogues in most of the investigated genomes might be due to their fragmented status as the majority of the data were sequenced using short-read sequencing methods. These technologies may not be adequate tools for the analysis of repeats. These facts hinder the full assembly of IS*Cfe1* and explain our relaxed BLAST coverage cut-off (>30%).

In order to investigate the overall sequence variability of IS*Cfe1*, we compared sequences from 123 IS*Cfe1* in which >70% (1,500 bp) of the sequence was obtained (Figure 3C and Supplementary Figure S1). 87 sequences were from strains with single copies and 35 sequences from seven strains with up to eight copies. Based on the sequence divergence of IS*Cfe1*, two stable and divergent groups exist (Figure 3C and Supplementary Figure S1). The mean SNP differences of 101 IS*Cfe1* sequences within group I was 1.73 SNPs (*P* distance = 0.003). The mean SNP differences of 22 IS*Cfe1* sequences within group II was 6.39 SNPs (*P* distance = 0.001). The mean SNP differences between both groups was 124.43 SNPs (*P* distance = 0.06). Interestingly, the two IS*Cfe1* groups were reflected by the phylogenetic structure of clade 1 (Figure 3B), as group I IS*Cfe1* clustered together in a subclade of clade 1, which is distant by 123 different genes to subclade 2. Subclade 2 harbors the group II of IS*Cfe1*, exclusively.

### MLST for *Campylobacter fetus* Subtyping

Multilocus sequence typing grouped the 283 strains into eleven sequence types (STs; Figure 2 and Supplementary Table S3). The most frequent MLST types in this study were ST4 (*n* = 93) and ST6 (*n* = 70). Seventeen strains were not assigned to known STs (Supplementary Table S3).

In clade 1, 88 out of 100 genomes were assigned to known STs, including 87 genomes of ST4 and one genome of ST70 (ERR1046003 [INTA19]). Twelve genomes were not reported to known STs. This includes one genome (ERR1203904 [UK13]) with a new variant (G259A) for the *gltA* gene as well as 11 genomes (10 of them form a cluster) that had 85–88% coverage of the *aspA* gene. In an attempt to improve the coverage for this *aspA* gene, we used FASTQ reads and directly mapped them against the *aspA* gene (Hunt et al., 2017). However, this has not improved the coverage pattern across the gene.

The genomes of clade 2 were assigned to ST6 except seven genomes that comprise one genome (ERR976358 [2006–367 h]) with a new variant (G222A) for the *uncA* gene as well as six genomes of ST4 (Figure 2 and Supplementary Table S3). The ST4 strains in clade 2 were previously reported in humans (Iraola et al., 2015), while two were of unknown origin. This is in contrast to ST4 in clade 1, which was reported in cattle. Interestingly, ST6 was just one allele distant to ST4. Similarly, clade 2 was very closely related to clade 1.

The phylogenetic clades 3 to 8 showed congruent results with MLST, in which clades 3, 5, and 8 corresponded to ST5, ST20, and ST69, respectively (Figure 2 and Supplementary Table S3). One strain in each of clades 4, 6 and 7 was not reported to known MLST type due to insufficient coverage to the MLST genes or due to the presence of gaps. The remaining strains of clade 4, 6, and 7 comprised ST2 (*n* = 21), ST11 (*n* = 15) and ST3 (*n* = 41), respectively.

In summary, MLST showed good agreement with the WGS-based phylogenetic clades, but WGS could resolve inconsistencies associated with bovine and human ST4 isolates. The ST4 was previously reported as *Cfv*-associated ST (Van Bergen et al., 2005a; Silveira et al., 2018) along with ST7 and ST12 (Van Bergen et al., 2005a). The latter were not detected in this study.

### *In silico* PCR Assays for *Campylobacter fetus* Subtyping

We tested 17 primer pairs for *C. fetus* including primer pairs from five PCR assays to detect *C. fetus*, one PCR to detect *Cff* (Wang et al., 2002), and 11 PCR assays to detect *Cfv*. Many PCR assays for the identification of *Cfv* target the IS*Cfe1* (Abril et al., 2007; Mcgoldrick et al., 2013; Van Der Graaf-Van Bloois et al., 2013), a *parA* gene (Hum et al., 1997; Mcmillen et al., 2006), but some assays have other targets (Van Bergen et al., 2005b; Moolhuijzen et al., 2009; Iraola et al., 2012; Figure 2 and Supplementary Table S3).

Results showed a high sensitivity of primer pairs that detect *C. fetus* at the species level, with primers developed by Mcgoldrick et al. (2013) being 100% sensitive. The primers reported by Abril et al. (2007) did not detect genomes that belong to clade 4 and clade 8, as well as some clade 1 genomes.

At the subspecies level, the *Cff-*specific PCR assay reported by Wang et al. (2002) was 32% sensitive to clade 2, 6, and 7, while genomes of the clades 1, 3, 4, 5, and 8 were all negative. On the other hand, the *Cfv-*specific PCR assays showed positive results only for clade 1 genomes, except one PCR primer-pair previously described by Moolhuijzen et al. (2009) and Iraola et al. (2012) that was also positive for genomes of clade 2 (*n* = 8 genomes) and clade 4 (*n* = 1 genome) (Figure 2 and Supplementary Table S3).

The IS*Cfe1* specific primer described by Mcgoldrick et al. (2013) was 100% sensitive to all genomes of clade 1 (Figures 2, 3 and Supplementary Table S3). One primer set (ISC2) described by Van Der Graaf-Van Bloois et al. (2013) was also 100% sensitive to clade 1 genomes (Figures 2, 3 and Supplementary Table S3). Nevertheless, IS*Cfe1* primers from Abril et al. (2007) as well as one primer set (ISC1) from Van Der Graaf-Van Bloois et al. (2013) could not detect most of group II IS*Cfe1-*carrying strains (*n* = 22 strains, Figures 2, 3 and Supplementary Table S3). The binding site of latter primer pairs located within a variable sequence region between the IS*Cfe1* group I and group II (Supplementary Figure S2). In contrast, PCR primers for the *parA* gene described by Hum et al. (1997) and Mcmillen et al. (2006) detected only 55 and 52% of clade 1 genomes, respectively. These results were confirmed using BLAST search (90% identity) for the *parA* gene (CP006999.2:1246091–1246231) in which we found the gene only in 55% of clade 1 genomes. We, therefore, concluded that IS*Cfel*-positive genomes do not necessarily carry the *parA* gene explaining the lack of congruence for different PCRs to detect *Cfv* (Figure 2 and Supplementary Table S3). Furthermore, the *in silico* evaluation of *C. fetus*-specific PCRs indicates that the primer pairs described by Mcgoldrick et al. (2013) performed best at the species and subspecies levels. This is in contrast to the updated international standards for the BGC at the OIE Terrestrial Manual (2018) (OIE) in which Abril et al. (2007) showed the highest sensitivity (97%) to detect *Cfv*. Yet, based on our analysis, this PCR assay was only 78% sensitive and could not detect 22 genomes with IS*Cfe1* group II of the investigated dataset.

Moreover, the sequence divergence (two groups) and repetition (multiple copies within the genomes) of IS*Cfe1* may hinder its correct identification with PCR assays as described (Van Der Graaf-Van Bloois et al., 2013). The results of *in silico* PCR may substantially differ from *in vitro* PCRs due to differences in primer binding. In addition, the fragmentation of most of the investigated genomes may negatively influence the output of *in silico* PCR. This can also be considered as a limitation of this study.

### New Markers and a Bioinformatics Tool for *Campylobacter fetus* Subtyping

We determined group-specific nucleotide markers for the eight defined clades, which could be investigated in the new strains for fast clade delineation without the need to reconstruct the whole phylogeny. These nucleotide markers were defined as specific nucleotide variants, which at a specific position, are identical in all genomes of one specific clade and different from all genomes of all other clades, ignoring indels and variants located in the intergenic regions or within repeated sequences (Supplementary Table S5).

Given the clinical importance of clade 1 being the cattle specific clade of this species, we identified eleven stable nucleotide markers specific for this clade, consequently for the subspecies *Cfv* (Table 2). The eleven SNPs comprised six synonymous and five non-synonymous variants and were identified within the most stable and strict core genes in all strains. The genes exhibited very limited nucleotide diversity with 4 to 13 variants observed for the eleven genes. These genes apparently have not recombined (non-significant PHI test) or had detrimental mutations that might have resulted in internal stop codons.

**TABLE 2:** Eleven characteristic nucleotide markers for clade I in comparison to other clades.

Nucleotide Position in reference genome	Locus tag	Protein ID	Gene Length	Gene	Product	Cfv (*n* = 100)	Cff (*n* = 182)	Effect to reference sequence	Cfv AA	Cff AA
39784	CFF8240_0048	ABK83415.1	396	*rpsH*	ribosomal protein S8	T	C	synonymous	L	L
343646	CFF8240_0373	ABK82476.1	552		conserved hypothetical protein	T	C	synonymous	L	L
399029	CFF8240_0424	ABK81969.1	708		conserved hypothetical protein	C	T	synonymous	G	G
535915	CFF8240_0528	ABK83346.1	1131		integral membrane protein-permease component, involved in lipoprotein release	T	C	synonymous	S	S
654050	CFF8240_0641	ABK82108.1	507		Hit family protein	A	G	non-synonymous	I	V
979346	CFF8240_0977	ABK82797.1	747		NADP-dependent l-serine/l-allo-threonine dehydrogenase ydfg	C	T	non-synonymous	C	Y
1020607	CFF8240_1016	ABK82995.1	879		phosphatase, Ppx/GppA family	A	C	non-synonymous	D	E
1025195	CFF8240_1023	ABK83085.1	615		translocator protein, LysE family	C	T	non-synonymous	S	N
1359201	CFF8240_1380	ABK83386.1	579		general glycosylation pathway protein	C	T	non-synonymous	M	I
1438083	CFF8240_1456	ABK82820.1	1032	*hemE*	uroporphyrinogen decarboxylase	A	G	synonymous	D	D
1452736	CFF8240_1473	ABK83285.1	1791	*lepA*	GTP-binding protein LepA	G	A	synonymous	T	T

**FIGURE 4:**
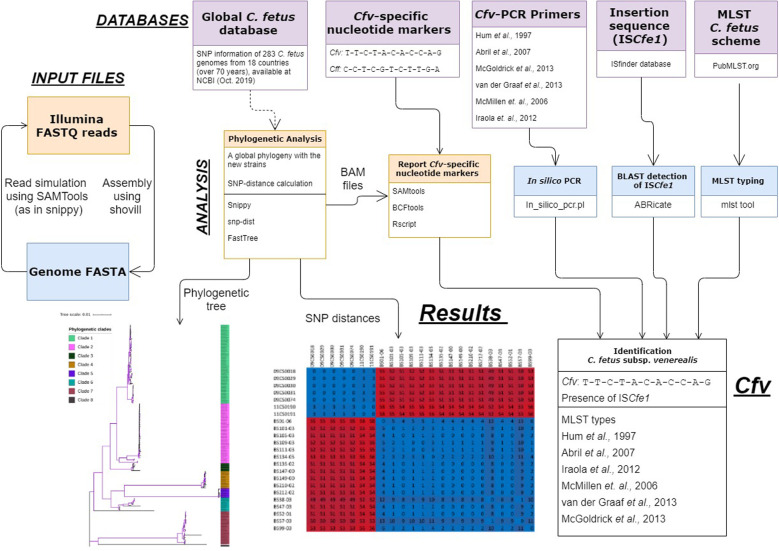
An overview of the *cfvCatch* workflow to identify the *Campylobacter fetus* subsp. *venerealis* (*Cfv*) strains.

We then developed “*cfvCatch*”^11^, a bioinformatics toolbox that fetches genomic data of *C. fetus* (Figure 4) and reports nucleotide markers specific for clade 1 (*Cfv*) and markers specific for clades 2–8 (*Cff*). In addition, *cfvCatch* reports MLST types and the presence of IS*Cfe1* as well as results of *in silico* PCR assays. Nevertheless, to report subspecies, *cfvCatch* takes only into account the phylogenetic position of *C. fetus* strains and the presence of the IS*Cfe1*. Strains are characterized as *Cfv* if they (1) harbor the insertion element IS*Cfe1* and (2) have the nucleotide variants specific for clade 1 (T-T-C-T-A-C-A-C-C-A-G), while strains that (1) do not harbor the insertion element IS*Cfe1* and (2) have the nucleotide variants specific for clades 2–8 (C-C-T-C-G-T-C-T-T-G-A), are reported as *Cff*. This assumption has been applied to 283 *C. fetus* genomes that represent diverse strains of global distribution (Supplementary Table S1) and sequenced at various read lengths using Illumina platforms. We further integrated the typing results of this global data set in *cfvCatch* for future phylogenetic comparison of new strains. The current implementation of the tool makes use of sequencing read mapping to a reference genome, therefore FASTQ reads are required as input. This is the only mandatory parameter for this software.

We assessed the performance of *cfvCatch* at different sequencing depths (10× to 200×) using an exemplary data set of 20 *Cfv* strains. The results showed that at a coverage depth of more than 30×, the subspecies detection was highly reproducible. However, the coverage depth of 10–20× resulted in failure in subspecies assignment for two (out of 20) genomes (Supplementary Table S6).

## Conclusion

In this study, we *in silico* compared molecular methods for *C. fetus* subspecies identification to the global reference phylogeny. As shown here and in previous studies (*in vitro*) (OIE; Van Der Graaf-Van Bloois et al., 2013, 2014), results from current PCR protocols were not completely congruent and some of the IS*Cfe1*-specific PCRs did not detect most of the group II IS*Cfe1* (Abril et al., 2007; Van Der Graaf-Van Bloois et al., 2013). MLST provided good discrimination as ST4 correlated with *Cfv* strains. However, *Cff* diagnosed as the causative pathogen for bacteraemia in some human cases (Iraola et al., 2015) also belonged to ST4. Additionally, we found different STs for strains that carry IS*Cfe1* and were positive in *Cfv*-PCR assays. These findings underline the lack of congruence for PCRs and MLST for *C. fetus* subtyping. It is noteworthy that the most common PCR system for *Cff* definition relies on the negative output of a *Cfv*-selective PCR assay. Negative samples for this PCR are regarded as *Cff*. This system of differentiation can lead to false-positive results.

Whole-genome analysis provides a very high resolution and can be reliably used to identify closely related strains for outbreaks and surveillances (Ardelean et al., 2019; Hsu et al., 2020). Whole-genome comparison defines a clonal structure (clade 1) for the IS*Cfe1-*carrying genomes. This clade was previously described by Iraola and colleagues and was identified as the cattle-specific clade of *C. fetus* in concordance with the epidemiology of *Cfv*. In addition, IS*Cfe1*, which is considered to be the *Cfv* marker sequence, was exclusively present in this clade. Furthermore, the molecular methods for subtyping the *C. fetus*, including MLST and PCRs, are not congruent, but showed positive results for clade 1, only.

In conclusion, we reinforce the importance of the clade structure initially described by Iraola and colleagues in 2017 for molecular subtyping of the *C. fetus* by adding 114 additional genomes. The strains of clade 1 harbor IS*Cfe1* and correspond to the subspecies *Cfv*, while strains of clades 2- 8 correspond to *Cff*, which do not harbor the IS*Cfe1* element. A PCR targeting IS*Cfe1* is recommended for subtyping *C. fetus*. In addition, we defined eleven nucleotide variants as specific markers for clade 1 and developed a bioinformatics toolbox available at https://gitlab.com/FLI_Bioinfo/cfvcatch for the fast identification and subtyping of *C. fetus* using whole genome sequence data based on the recently described clade designation.

## Data Availability Statement

The original contributions presented in the study are included in the article/Supplementary Material, further inquiries can be directed to the corresponding author.

## Author Contributions

MA-G designed the study, performed analysis and wrote the manuscript. HH, HT, and JL supervised the work and contributed to the interpretation of the data. All authors contributed to the drafting of the manuscript.

## Conflict of Interest

The authors declare that the research was conducted in the absence of any commercial or financial relationships that could be construed as a potential conflict of interest.

## Footnotes

1 https://github.com/tseemann/shovill

2 https://github.com/tseemann/snippy

3 https://github.com/tseemann/snp-dists

4 https://github.com/sanger-pathogens/snp-sites

5 https://github.com/rambaut/figtree

6 https://github.com/tseemann/mlst

7 https://github.com/egonozer/in_silico_pcr

8 https://www.geneious.com

9 https://github.com/tseemann/abricate

10 https://github.com/lh3/seqtk

11 https://gitlab.com/FLI_Bioinfo/cfvcatch

## Supplementary Material

Supplementary Figure 1 Cluster analysis of the IS*Cfe1* sequences showing the IS*Cfe1* sequence divergence as represented by a minimum spanning tree. Figure was created using BioNumerics version 6.6, in which the IS*Cfe1* sequences were treated as categorical character data, with a priority rule type set to “number of n-locus variants.”

Supplementary Figure 2 Alignment of the two sequence groups of IS*Cfe1* with highlighted primer positions.
