# Supplementary figures and images for: Phylogenomic Analysis of Campylobacter fetus Reveals a Clonal Structure of Insertion Element ISCfe1 Positive Genomes
Source: Front Microbiol. 2020 Nov 12;11:585374. doi: 10.3389/fmicb.2020.585374 (PMC7688749; doi:10.3389/fmicb.2020.585374)

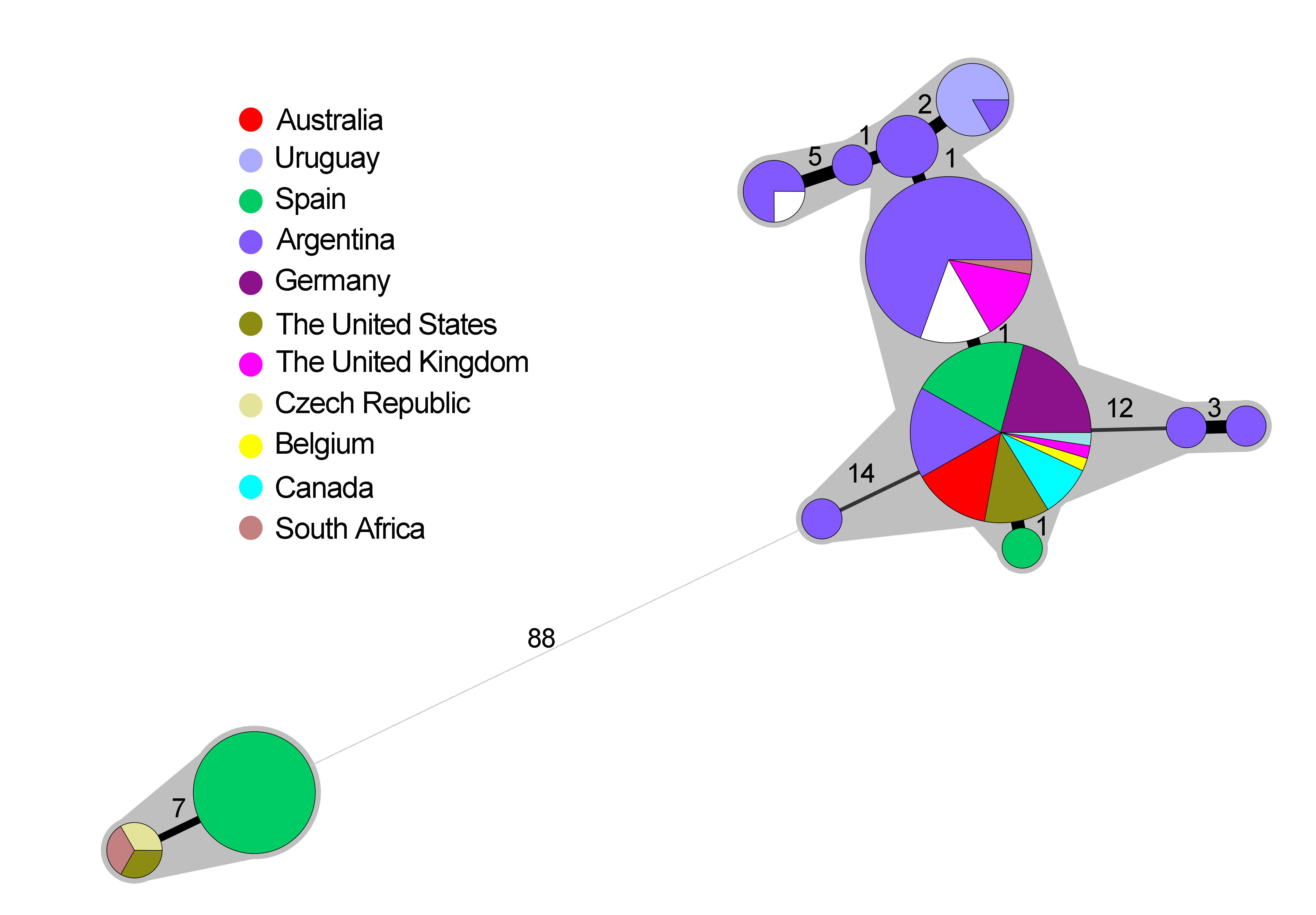

Supplement: Supplementary Figure 1 — Cluster analysis of the ISCfe1 sequences showing the ISCfe1 sequence divergence as represented by a minimum spanning tree. Figure was created using BioNumerics version 6.6, in which the ISCfe1 sequences were treated as categorical character data, with a priority rule type set to “number of n-locus variants.” [file Image_1.TIF]

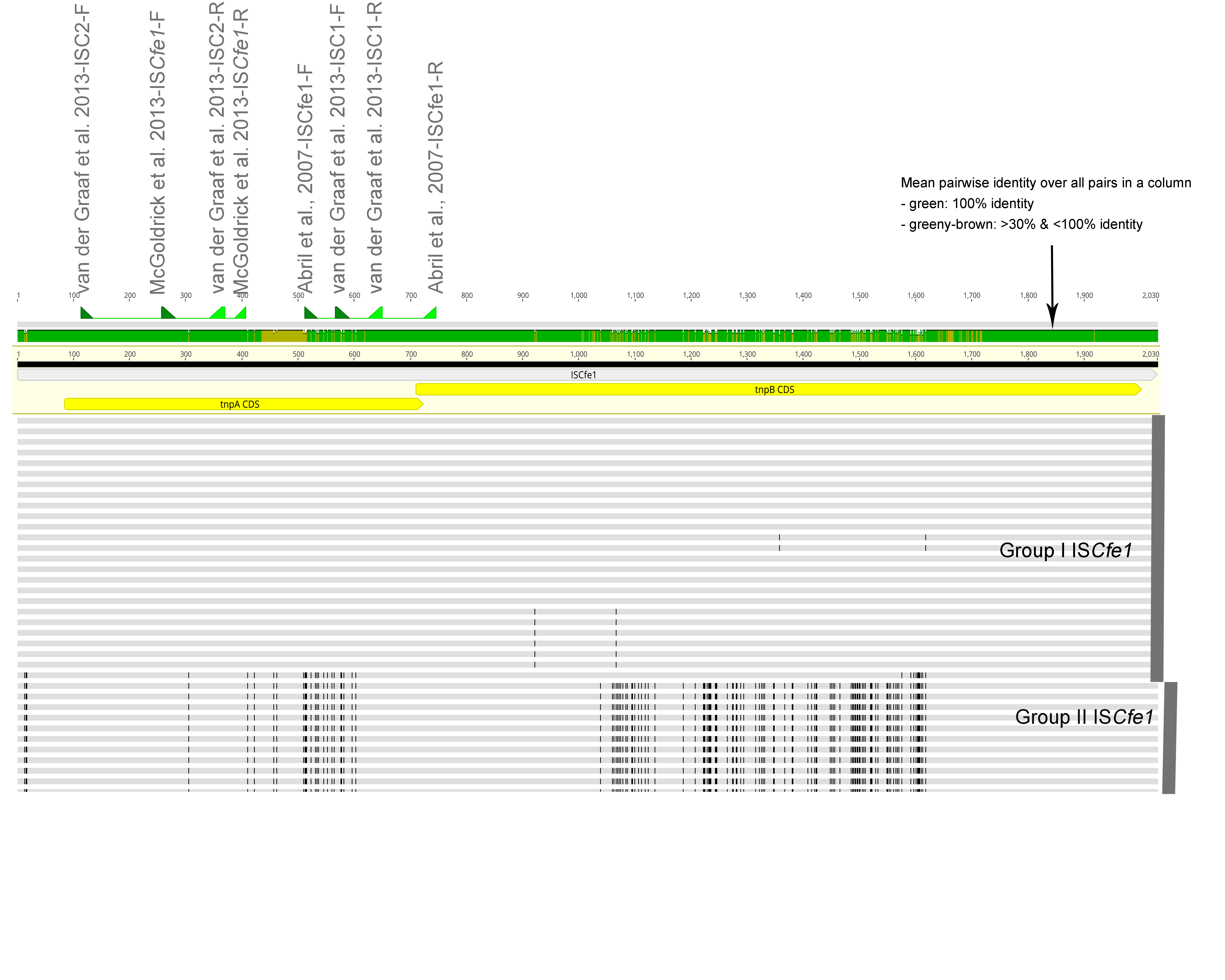

Supplement: Supplementary Figure 2 — Alignment of the two sequence groups of ISCfe1 with highlighted primer positions. [file Image_2.TIF]
